# Policy and legal perspectives on the traditional Chinese medicine varietal protection system (TCMVPS): reform or abolition?

**DOI:** 10.3389/fphar.2025.1724528

**Published:** 2026-01-06

**Authors:** Yanhui Wang, Ke Sun, Dong Hua

**Affiliations:** 1 School of Elderly Care Services and Management, Nanjing University of Chinese Medicine, Nanjing, China; 2 School of International Education College, Nanjing University of Chinese Medicine, Nanjing, China; 3 School of Health Economics and Management, Nanjing University of Chinese Medicine, Nanjing, China

**Keywords:** China, drug regulation, innovation incentives, intellectual property (IP), policy reform, traditional Chinese medicine varietal protection system (TCMVPS)

## Abstract

**Background:**

The Traditional Chinese Medicine Varietal Protection System (TCMVPS) was introduced in 1993 as a regulatory mechanism intended to safeguard key formulations, ensure product quality, and provide incentives for industrial development. After more than three decades of implementation, the system has played a role in stabilizing the market and preserving certain classic varieties. At the same time, its effectiveness and long-term relevance have become a matter of debate.

**Methods:**

This study reviews the legislative framework, implementation history, and current practice of the TCMVPS. It draws on policy documents, regulatory guidelines, and existing scholarship to examine how the system interacts with related regimes such as patents and drug registration. A comparative perspective is also adopted to highlight distinctive features and limitations. This study provides the comprehensive empirical and policy analysis of the TCMVPS’s evolution and reform prospects, contributing to the broader debate on sui generis protection of traditional medical knowledge.

**Results:**

The analysis shows that the system has contributed to protecting well-known TCM products and provided a degree of market exclusivity for approved varieties. However, several weaknesses have emerged: the narrow scope of protection, the ambiguous relationship with intellectual property law, the quasi-mandatory nature of applications under market pressure, and the reduced incentive for continuous innovation during the protection period. These challenges have limited the system’s capacity to balance protection with innovation and competition.

**Conclusion:**

The TCMVPS illustrates the opportunities and pitfalls of designing sui generis mechanisms for traditional medicine protection. Its future relevance depends on substantive reform, including clearer alignment with intellectual property and drug regulatory frameworks, expansion of eligible categories, and the incorporation of mechanisms that encourage continuous clinical and technological improvement. Beyond China, the experience of the TCMVPS provides valuable lessons for other jurisdictions seeking to reconcile traditional knowledge preservation with modern regulatory and innovation systems.

## Introduction

In contemporary industrialized and information-driven societies, the patent system is widely acknowledged as one of the principal institutional mechanisms for promoting innovation in the pharmaceutical field (Correa, 2000; Maskus, 2000).

Both in traditional Chinese medicine (TCM) and in Western biomedicine, patent protection has frequently been regarded as the standard legal route for safeguarding research outcomes. The rationale rests on the disclosure requirement embedded in the patent regime: by compelling applicants to reveal technical details, patents are designed not only to grant exclusive rights to inventors but also to contribute to the wider dissemination of knowledge, thereby facilitating processes of technological diffusion (Dutfield, 2017). In practice, patents have increasingly been used as a proxy for innovation capacity. Modern pharmaceutical enterprises tend to evaluate their own competitiveness, as well as that of their rivals, on the basis of both the quantity and the quality of patents acquired (World Intellectual Pro perty Organization, 2020).

Nevertheless, the structural characteristics and inherent design of the patent system also create significant limitations on its applicability. As a legal institution rooted in modern intellectual property frameworks, the patent regime often struggles to accommodate knowledge systems and products that originate in pre-modern or non-Western traditions, with TCM representing a particularly illustrative example (Roffe et al., 2006). Scholarly debates have repeatedly emphasized that the standardized criteria of patentability—such as novelty, inventive step, and industrial applicability—are frequently misaligned with the collective, practice-based, and historically accumulated nature of TCM knowledge (Lin et al., 2018). These ongoing discussions underscore persistent shortcomings in the current patent mechanism with respect to its capacity to provide effective and appropriate protection for TCM resources and innovations (Gervais, 2005; Qi et al., 2009; Li, 2024).

Within China’s legal protection framework for TCM, the TCM Varietal Protection System (TCMVPS) serves as an important institutional mechanism designed to complement patent law. The system classifies and grants protection to proprietary Chinese medicines based on their demonstrated therapeutic efficacy. By prioritizing “practical utility” while easing the strict requirement for “novelty,” the TCMVPS provides a legal pathway that partially addresses the limitations of the conventional patent system in safeguarding TCM knowledge. Over the more than three decades since its introduction, the system has contributed to the growth of China’s TCM industry and has supported the ongoing development and commercialization of innovative TCM products.

Nevertheless, as TCM undergoes accelerated globalization and technological modernization, the limitations of the TCMVPS have become increasingly apparent. Being a regulatory framework unique to China, the system exhibits a strong administrative protectionist orientation, which has at times complicated its integration with broader intellectual property (IP) regimes. This structural misalignment reflects a wider tension between China’s indigenous institutional design and internationally recognized IP norms, prompting ongoing scholarly and policy discussions regarding potential reforms and the system’s long-term viability.

## Methodology

This study employs a combination of normative legal analysis and comparative research to examine the evolution, functions, and reform trajectory of the TCMVPS. Three complementary approaches support this analysis.

First, a normative legal analysis was conducted. The primary materials analyzed include the 1993 *Regulations on the Protection of TCMVPS* (hereinafter “the *Regulations*”) and subsequent official interpretations and evaluation guidelines. Through close reading of these normative documents, the study identifies the internal mechanisms of TCMVPS in terms of institutional structure, legal logic, and functional positioning.

Second, a comparative policy analysis was carried out to examine TCMVPS alongside the patent system. By comparing their scope, authorization criteria, protection terms, and applicability to traditional medicine varieties, the study identifies areas of functional overlap, institutional gaps, and coordination challenges, providing a basis for understanding reform needs.

Third, to capture the system’s practical implementation, publicly available literature was analyzed, including legal and policy texts, official statistics and reports, case materials, and relevant commentaries. These sources offer an empirical foundation to trace the system’s operational trends over time.

## Establishment background

### Establishment of the TCMVPS

China’s system for protecting varieties of TCM dates back to 1993, a period when the domestic production of proprietary TCM formulations was plagued by widespread imitation and redundant manufacturing of identical products. For example, *Angong Niuhuang Wan*—a TCM preparation used for heart stroke, coma, and febrile conditions, with key export markets such as Indonesia, Vietnam, and Singapore—was then manufactured by more than 100 producers in China (Weber Consulting, 2025). Similarly, *Compound Danshen Tablets*, another formulation popular abroad for treating cardiovascular and cerebrovascular diseases, were produced by hundreds of pharmaceutical enterprises (Su et al., 2016). Such low-level replication severely infringed upon the legitimate interests of originator TCM manufacturers (the then marketing authorization holders MAHs), discouraged their incentives for R&D, and compromised the quality and reputation of related products. To standardize the market and improve the quality of TCM formulations, the State Council of China initiated the drafting of the *Regulations* in 1991, introducing an administrative protection mechanism for high-quality TCM products. The *Regulations* entered into force on January 1, 1993. According to the *Regulations*, the TCMVPS operates on a voluntary application basis, the MAHs for TCM products (initially mainly manufacturers of proprietary Chinese medicines) may apply for Class I or Class II protection based on the therapeutic functions and demonstrated efficacy of the product in question (see Table 1).

**TABLE 1:** Comparison between class I and class II of TCMVPS.

Dimension	Class I protection	Class II protection
Protection conditions	1) Demonstrates special therapeutic efficacy for specific diseases 2) Artificially manufactured products equivalent to nationally class I–protected wild medicinal species 3) Used for the prevention and treatment of special diseases	1) Varieties that meet class I protection conditions or have been released from class I protection 2) Demonstrates significant therapeutic efficacy for specific diseases 3) Active substances extracted from natural medicines and special formulations
Protection term	Thirty years, 20 years, or 10 years (depending on the variety)	Seven years
Extension of protection	Applications for extension may be filed within 6 months before expiration; each extension shall not exceed the duration of the initial approval	Upon expiration, an extension may be applied for; each extension period is 7 years
Confidentiality requirements	The formulation and manufacturing process must remain strictly confidential during the protection term and shall not be disclosed	No explicit confidentiality requirement
Overseas transfer	Transfer of formulation and manufacturing process must comply with national confidentiality regulations	No special confidentiality requirements for transfer
Penalties for disclosure	Individuals responsible for disclosure shall be subject to administrative sanctions; if the act constitutes a crime, criminal liability shall be pursued	No specific penalty provisions for disclosure

For proprietary Chinese medicines that demonstrate major breakthrough efficacy in treating specific diseases or are used to prevent or treat special diseases, Class I protection may be granted. The protection period can be 10, 20, or 30 years (renewable once). During the protection period, the product’s formula and manufacturing process are kept confidential and are accessible and usable only by the applicant.

For proprietary Chinese medicines that demonstrate significant clinical advantages or superior efficacy compared to other similar products in treating a specific disease, syndrome, or symptom, Class II protection is available. The protection period is generally 7 years (renewable once). Since the formulas and processes of Class II protected TCM products are usually already open in the public domain, protection is implemented through an administrative licensing system. Under this system, the government authorizes qualified TCM manufacturers to produce the protected product—typically limiting authorization to no more than ten manufacturers. Manufacturers without the required qualifications are not permitted to produce the protected variety.

### Content of the TCMVPS

In terms of institutional design, the TCMVPS shares some similarities with globally recognized plant variety protection systems (PVPS), particularly regarding IP safeguards and mechanisms that incentivize innovation. Under the *International Convention for the Protection of New Varieties of Plants* (UPOV), the PVPS primarily aims to protect breeders’ rights by granting them exclusive rights over the production, sale, and importation of their new plant varieties (UPOV, 1991). These provisions help ensure that technical achievements are not misappropriated or disclosed without authorization, thereby safeguarding the legitimate interests of the breeders.

Similarly, the TCMVPS—particularly its Class I protection—places strong emphasis on granting exclusive rights over technical information and data involved in the research, development, and production of proprietary Chinese medicines. Functionally, the system is designed to restrict the unauthorized use or disclosure of sensitive technical knowledge, thereby stimulating continued innovation within the TCM industry. Rather than merely representing an attempt to strike a balance between IP-style exclusivity and the safeguarding of traditional medical knowledge, the institutional logic of the TCMVPS is better understood as an adaptation to China’s specific regulatory context and the distinctive characteristics of TCM, rather than a direct transplantation of the PVPS. (see Table 2).

**TABLE 2:** Comparison of three regimes.

Feature dimension	Traditional Chinese medicine varietal protection system	Plant variety protection system	Patent system
Protected subject matter	Medicines (end products and technical solutions)	Living organisms (plant varieties)	Technical solutions
System attribute	Quasi-intellectual property right	Intellectual property right	Intellectual property right
System objective	Improving industry standards, safeguarding public health, and incentivizing innovation	Encouraging breeding innovation and supporting agricultural and forestry development	Stimulating technological innovation across all fields
Focus of rights	Market access control and exclusive rights in commercialization	Exclusive rights in the commercialization of propagating materials	Exclusive rights in implementing technical solutions

In accordance with the *Regulations* and the *Items and Instructions for Application Materials for TCMVPS* (issued by the National Committee for Evaluation of TCMVPS and effective as of January 1, 2012), the core materials required for a Class I protection application include:

Basic drug information: name, composition, dosage form, formulation, and manufacturing process (key steps must be specified, though not in full detail); Quality standards: including quality control methods for raw materials, intermediates, and finished products (e.g., chromatographic fingerprint profiles, assay of characteristic components); Clinical research data: evidence supporting the drug’s safety and efficacy (such as clinical trial reports and literature reviews); Research data on the production process: demonstrating the rationality, stability, and reproducibility of the process.

During the actual review, all critical process parameters must be submitted to the drug regulatory authority, but are kept confidential and not publicly disclosed.

Compared to the UPOV system, the TCMVPS targets the “medicinal product” itself, focusing primarily on the formula, manufacturing process, and efficacy of the proprietary Chinese medicine, rather than the breeding process of the medicinal raw materials. Nevertheless, if the raw materials of a TCM variety involve artificial propagation techniques for rare or endangered wild medicinal resources (e.g., ginseng seed treatment) and such techniques are critical to the product’s efficacy, relevant propagation technical data should also be submitted. Such information generally falls under commercial secrets and may be submitted under a confidentiality request to limit disclosure—for example, by providing it only to the reviewing authority without public release.

To date, only a limited number of proprietary Chinese medicines have been granted Class I protection. Representative examples include *Yunnan Baiyao*, *Zhangzhou Pien Tze Huang*, D*ong’e Ejiao*, and *Liushen Pills*, all of which are regarded as possessing notable clinical effectiveness. Because the core technologies of these products were never patented, their MAHs have been able to preserve the relevant knowledge through confidentiality measures, rather than disclosure through the patent system.

The design of the TCMVPS takes into account not only technical and industrial factors but also the geographical characteristics of medicinal materials and regional cultural traditions. These considerations are particularly evident in the mechanism of Class II protection. Unlike Class I protection, which is characterized by strict exclusivity and confidentiality of prescriptions and production processes, Class II protection does not impose secrecy obligations. Under this model, both the original developer and other qualified enterprises may apply for production authorization of the same protected variety, provided they comply with the relevant regulatory standards. Production rights are granted through administrative licensing, which sets clear benchmarks for quality, safety, and standardization. Only those enterprises that receive official approval are permitted to manufacture the protected medicine, while unauthorized production constitutes a violation subject to administrative penalties. In essence, Class II protection functions as a form of government regulation over the commercialization of proprietary Chinese medicines. By restricting the number of authorized producers, the system helps to avoid redundant production, mitigate market disorder, and maintain stable industrial competition.

More broadly, the TCMVPS was created to safeguard proprietary of Chinese medicine and has played a crucial role in curbing unauthorized imitation of well-established herbal prescriptions. At a time when no international consensus had yet been reached on the legal protection of “traditional medical knowledge”, the system represented China’s *sui generis* approach to IP. By combining certain international practices with locally embedded features, the TCMVPS contributed to expanding the market presence of innovative TCM products and promoted the modernization, industrial intensification, and scaling-up of the sector (Jiang et al., 2020). Moreover, the experience accumulated through this system provided valuable insights for the subsequent introduction of other IP mechanisms in China, such as geographical indications applied to medicinal materials, thereby reinforcing the institutional framework for the protection of traditional medicine resources (Chen et al., 2013).

### System operation status

#### Review of the operation process

The *Regulations* have been in effect in China for thirty-two years. According to statistics from announcements accumulated by the National Medical Products Administration (NMPA), as of the end of August 2025, the Chinese government had issued a total of 4,681 TCMVPS certificates (including both initial and renewed protection varieties), covering 3,118 announcements for new protections, 1,561 announcements for protection extensions, and 2 announcements for termination of protection.

Of these, 10 varieties from 10 enterprises have obtained Class I protection; 1,807 varieties from 1,669 enterprises have received Class II (initial) protection, accounting for approximately 16.5% of all proprietary Chinese medicines in China; and 978 varieties from 871 enterprises have been granted renewed protection.

**FIGURE 1:**
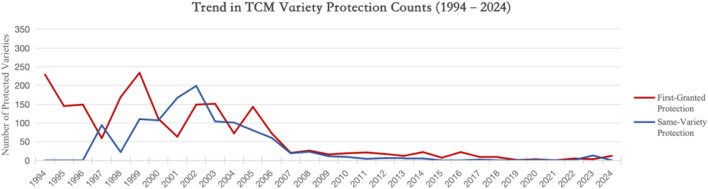
Trend in the Number of Protected TCM products of 1994–2024. (Data source: Announcements of TCMVPS on the website of the NMPA, with the statistical year based on the variety number).

By objectively improving the quality of TCM formulations, the system encouraged active participation from TCM area. Since its implementation, the application of the TCMVPS has evolved through the following stages. (see Figure 1).

The first protection period (1993–2001). During this phase, a large number of traditional proprietary Chinese medicines—such as *Fuzi Ejiao*, *Longmu Zhuanggu Granules*, *Niuhuang Shedan Chuanbei Liquid*, and *Anshen Bunao Liquid*—were applied for and granted Class I or Class II protection. On average, about 145 varieties received initial protection each year. Announcements for renewed protection began to be issued in 2000, and a total of 207 varieties, mostly exclusive formulations, were granted protection extension in 2000 and 2001.

From the perspective of protection targets, this phase centered on maintaining the confidentiality of key formulations and production techniques for proprietary Chinese medicines. However, as the system was still in its initial establishment and implementation stage, the application procedures and review standards were relatively vague and lacked transparent mechanisms.

The Regulatory Adjustment Period (2002–2008). During this phase, the protected TCM products began to exhibit signs of structural optimization and reduced volatility in their distribution. The number of initial protections decreased to an annual average of approximately 88, representing a decline of roughly 40% compared with the average of 145 per year observed during the first protection period. Announcements for renewed protection became more regular from 2000 onward, with a cumulative total of about 546 issued between 2002 and 2008, averaging 78 per year. This trend indicates a more systematic approach to extending the market lifecycle of protected TCM products.

The number of same-variety protections peaked in 2005–2006, with 195 and 281 announcements, respectively, before declining thereafter to 144 by 2008. This pattern reflects increasingly stringent oversight and the implementation of more standardized application management procedures. Although the number of terminated protections fluctuated over this period, it generally averaged around 100 per year, which largely corresponds to the natural expiration of protection periods.

Throughout this regulatory adjustment phase, the TCMVPS was applied in a more standardized and methodical manner, contributing to further improvements in the quality and market competitiveness of proprietary Chinese medicines. Many MAHs responded by increasing research and development investment and launching a range of new, market-competitive varieties. Additionally, some of the protected TCM products began to access international markets, signaling the gradual globalization of certain proprietary formulations.

Nevertheless, the system continued to encounter challenges. A notable concern was its inadequate linkage with the patent system, which limited the integrated protection of TCM innovations. Moreover, the lack of a dynamic update mechanism for already-protected proprietary Chinese medicines has resulted in relatively sluggish technological advancement, causing some protected TCM products to fall behind in modernization and innovation despite holding formal protection status.

The Period of Institutional Dormancy and Functional Weakening (2009–2024) During this phase, the number of applications for TCMVPS declined sharply, and overall system activity fell to historically low levels. Both new protections and renewed ones dropped dramatically, averaging only about 5–10 approvals per year, far below the levels observed in earlier periods. The implementation of same-variety protection nearly stagnated, while the number of terminations also decreased. The nadir occurred between 2015 and 2016, when only 26 new protected TCM products were approved annually, highlighting a pronounced decline in the system’s vitality and operational engagement.

This decline was primarily attributable to the heightened thresholds for application and the increased difficulty in obtaining protection, yet the expected protective effects were not fully realized. After 2017, there was a modest recovery in applications, but overall enthusiasm remained subdued due to anticipated amendments to the *Regulations* and ongoing industry adjustments. It was not until 2022, when the draft amendment of the *Regulations* was released for public comment, that new approvals rose to 16, followed by a further increase to 12 in 2024, indicating an initial but tentative recovery of system activity.

The TCMVPS was initially established within the framework of China’s traditional pharmaceutical administrative system. During that time, medicinal products had not been included as patentable subject matter under Chinese law. However, following the 1993 revision of China’s Patent Law, the pharmaceutical patent system was formally established, and patent applications gradually became the primary mechanism for protecting the research, development, and production of all drugs, including TCM products. Compared with the TCMVPS, patent protection is more consistently aligned with internationally recognized IP standards and better accommodates the requirements of an industrialized pharmaceutical sector. Consequently, applications for TCMVPS have steadily declined over time. This trend presents a persistent dilemma for the system: raising application requirements further could exacerbate the reduction in application numbers, whereas relaxing standards might weaken the system’s capacity to incentivize and safeguard innovation in proprietary Chinese medicines, thereby challenging the original intent of the system’s establishment.

#### Practical needs and controversies in system operation

The TCMVPS is inherently voluntary, with applications dependent on the decisions of MAHs. In practice, however, if the holder fails to submit an application for TCMVPS in a timely manner, other TCM enterprises can readily engage in “free-riding,” thereby undermining the original holder’s competitive advantage. Even in situations where certain TCM products have already been granted patents, preemptive production by competitors continues to occur, illustrating persistent challenges in safeguarding proprietary formulations.

Although the relevant regulations state that patented proprietary Chinese medicines are governed by the Patent Law and do not require additional TCMVPS, MAHs frequently choose to pursue both types of protection simultaneously. This practice is primarily motivated by the need to secure production approval and to prevent competitors from obtaining production rights ahead of time, reflecting the critical importance of market exclusivity and commercial security.

The TCMVPS has effectively acquired a quasi-mandatory character, compelling MAHs to apply for protection and bear the associated administrative costs in order to maintain their production qualifications. In this context, the system has not fully realized its intended role of compensating for the limitations inherent in TCM patent protection. Instead, it introduces an additional layer of regulatory complexity, making TCMVPS a practical and administrative “burden” for holders.

In recent years, discussion and debate regarding whether to retain or abolish the TCMVPS have intensified. Critics argue that its weak integration with the patent system and the absence of analogous regimes in other countries of the world have hindered the international collaboration and cross-border cooperation in the pharmaceutical sector (He and Ma, 2023).

Nevertheless, it is undeniable that the difficulties associated with securing and maintaining TCM patents provide a tangible justification for the continued existence of the TCMVPS. The conceptual distinction between TCM and Western medicine in China arose alongside the introduction of modern Western medical science. Originally, “Western medicine” referred specifically to drugs imported from the West, though the modern definition has broadened to include all chemical drugs intended for human therapeutic use. TCM, by contrast, encompasses formulations developed according to traditional principles such as “理法方药” (theory, method, formula, and materia medica) and the compatibility rule of “君臣佐使” (sovereign, minister, assistant, and envoy), typically employing compound formulas that integrate multiple medicinal ingredients.

From the perspective of TCM theory, therapeutic effects within the human body rarely originate from a single herb or an isolated active ingredient. Rather, efficacy derives from the synergistic interactions among multiple components. Once processed into finished products, the chemical composition of TCM becomes increasingly complex. Conventional chemical and analytical terminology suitable for Western drugs is largely inadequate to accurately describe the structure and composition of proprietary Chinese medicines. Even when certain active ingredients can be quantified, such measurements alone cannot reliably predict or evaluate the overall therapeutic efficacy of a TCM formulation. Moreover, innovation in TCM is often incremental, typically producing modest improvements rather than substantial, breakthrough advances. Consequently, in comparison to the relatively streamlined patenting process for Western pharmaceuticals, TCM patent applications encounter significant challenges in meeting the standards of novelty and inventive step. The patent grant rate for TCM is therefore relatively low, and even when patents are awarded, protection is often limited to improvements in dosage forms or newly identified uses, resulting in a patent lifespan that is generally short (Liu et al., 2015; Liu et al., 2023).

Under these conditions, the return on investment for TCM developers remains comparatively limited. Given the substantial financial and temporal resources required for TCM research and development, the scarcity of effective legal protection has created an industry environment in which imitation can often be more commercially rewarding than original innovation.

The large-scale production of TCM formulas is realized through their transformation into proprietary Chinese medicines. Before these products enter the market, the primary legal challenge they encounter concerns the choice of an appropriate protection strategy. Patent protection is typically the first option considered by manufacturers, yet it often proves difficult in practice. From the perspective of technical protection, the more precisely an invention is described, the stronger the resulting patent protection will be. However, because the mechanisms of action of traditional formulas cannot be fully articulated in modern chemical or physical terms, they frequently fail to meet the statutory standards for patentability. Even when a patent is successfully granted, protection remains limited: uncertainties regarding the active substances or possible adjustments to the composition of multi-herb formulas mean that the patent is rarely ironclad. In other words, there is always room for others to develop an “improvement patent,” which is precisely what many MAHs fear—the act of disclosure does not necessarily secure meaningful protection.

In recent years, efforts to improve the success rate of patent applications for TCM have shifted toward isolating active compounds or active fractions. This approach, however, has attracted growing criticism. Research that focuses on extracting “effective substances” is often seen as treating traditional medicines merely as raw materials for new drug development rather than as therapeutic systems in their own right. In particular, when isolated compounds cannot replicate the therapeutic effects of the original formula, they may be patentable yet hold limited market value. Against this backdrop, the establishment of the TCMVPS has offered an alternative route for protecting Chinese medicinal products.

Compared with the patent system—where protection centers on the molecular structure of innovative drugs and requires inventions to satisfy the criteria of novelty, inventiveness, and utility—TCMVPS prioritizes “distinctive techniques plus clinical value.” Its protection targets mainly traditional multi-herb formulations, an area that patents struggle to cover. In this sense, TCMVPS focuses on the overall protection of standardized, effective, and quality-controlled proprietary Chinese medicines already on the market. Its core purpose is not to promote “innovation” in the patent-law sense, but rather to advance industrial policy objectives and broader public interests.

The modes of protection also differ significantly. Patent protection grants exclusive market rights in exchange for disclosure, relying on technical barriers to secure monopoly power. By contrast, TCMVPS operates more like a form of “premium certification” or a selective entry mechanism. Its value orientation lies in identifying and supporting high-quality products in the industry rather than safeguarding inventive concepts, thereby forming a complementary relationship with the patent system. Through a model of administrative confirmation combined with ongoing supervision, TCMVPS incorporates industry-specific criteria—such as the maturity of clinical application—with the aim of enhancing the quality, safety, and effectiveness of proprietary Chinese medicines.

In terms of protection duration, pharmaceutical invention patents provide a uniform 20-year term. TCMVPS, however, bases its protection period on the demonstrated therapeutic effect of the medicine and allows for extensions. This offers long-term protection for classical formulations that require continuous investment and maintenance, aligning more closely with the long historical cycle and developmental characteristics of TCM.

Taken together, these features show that the TCMVPS is better understood as a governance instrument rather than merely a quasi-IP right. The system still holds considerable potential for future development. At the same time, its practical operation has revealed structural vulnerabilities, and the recent decline in applications indicates that certain aspects of its design remain inadequate. These issues point to the need for functional improvements, not grounds for abolishing the system. Accordingly, future reforms of TCMVPS should focus on clarifying protection thresholds, enhancing coordination with the IP regime, and strengthening technical evaluation mechanisms.

### Exploration of institutional reforms

In response to these controversies, China’s National Administration of TCM (NATCM) and the NMPA have undertaken reforms to improve the TCMVPS since 2021. In 2022, the NMPA released a draft amendment to the *Regulations* for public consultation, proposing revisions to system provisions. Despite this progress, the draft amendment remains unenacted, primarily due to persistent ambiguities in the reform’s substantive direction—particularly regarding whether the TCMVPS should prioritize cultural heritage preservation or align with international patent frameworks. For the system to operate effectively, clarifying its relationship with the TCM patent system and drug registration procedures is essential; failure to do so risks undermining its long-term viability.

#### Enhancing the protection pathway: Integrating the TCMVPS with IP systems

The key to enhancing the effectiveness of the TCMVPS lies in clearly defining its unique functional positioning relative to established IP systems, particularly the patent system.

Some scholars have suggested that the patent system and the TCMVPS can be applied in tandem. That is, MAHs may pursue both forms of protection concurrently, utilizing patents to safeguard the product’s formula while leveraging the TCMVPS to secure market exclusivity (He and Ma, 2023). However, in practice, the substantive requirements under the TCMVPS differ from those imposed by the patent system. The TCMVPS places greater emphasis on the therapeutic functions, clinical indications, and practical applications of the TCM products, and in some cases permits protection for incremental improvements to existing technologies or drugs that are already publicly disclosed, as exemplified by Class II protection. Additionally, the TCMVPS does not strictly differentiate between the original developer and the manufacturer of a TCM variety, allowing for broader application of rights.

Consequently, when the proprietary Chinese medicinal products that have already been granted patent protection are simultaneously included within the TCMVPS, the same underlying technology becomes subject to overlapping regulatory frameworks. This overlap can easily result in unclear boundaries of rights, potential conflicts between protections, and issues of duplicate coverage, complicating both enforcement and compliance.

To achieve functional complementarity between the TCMVPS and the patent system, it is essential first to delineate the respective scopes of these protections. The TCMVPS should be primarily reserved for proprietary Chinese medicines that have not obtained patent protection, or for products that, due to technical characteristics or prior public disclosure, cannot meet the criteria for patentability yet retain substantial clinical value. The MAHs or the manufactures should be able to select the appropriate level of protection based on the product’s demonstrated efficacy and safety, thereby securing market exclusivity or access advantages for a reasonable period.

Conversely, proprietary Chinese medicines that have already been granted patent protection under applicable laws should rely primarily on the patent system, and should not be required to seek concurrent TCMVPS. This approach minimizes institutional redundancy and ensures that policy incentives remain balanced and effective.

When choosing an appropriate legal mechanism for protecting TCM products, a key consideration is the nature of the subject matter itself; different types of knowledge require different forms of protection. From a technical standpoint, innovations involving new compounds, active ingredients, preparation methods, or technologies that can be reverse-engineered are generally better suited for patent protection. However, the patent model—built on “disclosure in exchange for protection”—is often less effective for technologies whose competitive advantage depends on confidential formulations or unique processing methods. Such technologies include: compound TCM formulas whose complexity makes their critical features difficult to uncover through reverse engineering; intricate processing methods for decoction pieces and proprietary TCM products; certain technical know-how that may not be highly inventive but remains opaque to outsiders; and short-cycle technologies that evolve quickly. These kinds of knowledge are better protected through first-class protection mechanisms. In essence, the TCMVPS offers a form of “regulated confidentiality,” in which MAHs submit core data and processes to the regulatory authority in exchange for market protection, without being required to disclose the information publicly.

Moreover, to prevent an “institutional vacuum” following the expiration of protection periods, a transitional mechanism should be established linking the TCMVPS with IP systems. Such a mechanism would guide rights holders in adjusting their protection strategies in a timely manner prior to the expiration of protected varieties, thereby facilitating a smooth transition from administrative protection to formal IP-based legal safeguards.

In the practical operation of TCMVPS, the protection strategy of Yunnan Baiyao offers a representative example. Its powder and capsule products—both of which rely heavily on confidential formulations—were granted first-class protection in 1995 and successfully renewed in 2015. After more than three decades of market development, they have become the company’s flagship products. According to industry statistics released in 2024, these two products have long held leading positions in the national retail market for musculoskeletal and trauma-related proprietary Chinese medicines.

Building on this foundation, the company has developed modern dosage forms such as plasters, tinctures, and sprays, which are primarily protected through patents. In this way, the confidential-formula protection offered by TCMVPS and the disclosure-based protection of patents complement each other within the firm’s product portfolio: the TCMVPS safeguards the core, non-reverse-engineerable formula and processing techniques (“know-what”), forming the system’s foundational layer and ensuring continued exclusivity over traditional knowledge; patents then protect the specific product forms and application technologies built around that core (“know-how”), creating an outer protective barrier that supports dosage-form innovation, technical improvement, and value-chain expansion. Together, they form a multi-layered and coordinated protection framework that enables traditional medical knowledge to achieve continuous innovation within the modern IP systems.

### Expanding the scope of the TCMVPS: protection of processing techniques and classical formulations

A central debate concerning whether to retain or abolish the TCMVPS revolves around its currently limited protective scope. At present, the system provides coverage solely for finished proprietary Chinese medicines, leaving out the preparatory techniques and processing methods applied to the raw herbal materials used in these products. This narrow scope is widely considered to restrict both the practical utility and the overall effectiveness of the system, raising concerns among scholars, regulators, and industry stakeholders alike.

From a practical standpoint, the processing methods of Chinese medicinal materials are critical determinants of the quality, safety, and therapeutic efficacy of the final TCM products. The knowledge embedded in these techniques significantly influences product stability, clinical outcomes, and the reproducibility of therapeutic effects. Therefore, expanding statutory protection to include these preparation techniques could represent a meaningful step toward enhancing the system’s relevance and practical impact.

In recent years, calls have increasingly emerged to incorporate the processing techniques of Chinese medicinal materials within the scope of TCMVPS (Kang et al., 2023). These techniques, which have been honed over decades of clinical and practical experience, are fundamental to achieving therapeutic mechanisms such as “减毒增效” (reducing toxicity while enhancing efficacy)and “以毒攻毒” (using toxins to combat toxins). Over time, the accumulated expertise in processing has evolved into an independent discipline within Chinese pharmaceutical science, reflecting both empirical knowledge and methodical innovation.

However, these practical innovations often fail to meet the “inventiveness” requirement imposed by the patent system. Consequently, they are highly susceptible to rejection during patent examinations. In addition, the mandatory disclosure obligations associated with patent applications pose a risk of revealing proprietary technical information. As a result, many MAHs including pharmaceutical manufacturers, prefer to retain these techniques as confidential know-how rather than seeking patent protection, thereby safeguarding critical commercial interests.

To address the structural gaps identified above, several institutional reforms to the TCMVPS should be considered:

Broaden the scope of protectable subject matter. Techniques involving specific processing or preparation methods that have a substantive impact on therapeutic efficacy should be recognized as an independent category eligible for protection. A corresponding “first-class process protection” mechanism could be established to mirror the hierarchical structure applied to TCM varieties.

Introduce a confidentiality-based process recognition mechanism. Applicants should be allowed to submit sealed information on key processing steps for recordation with the pharmaceutical regulatory authority. This would enable the authority to assess the creativity and distinctiveness of the technique without requiring public disclosure of the detailed manufacturing process.

Adopt grant criteria that link technical assessment with industrial value. Applications for process protection should be supported by clinical evidence, data on pharmacological changes, and quality analysis results, ensuring that decisions are grounded in transparent and verifiable standards.

Through these targeted reforms, the TCMVPS would be better positioned to incorporate traditional experiential techniques within its existing institutional framework, thereby providing stronger support for the innovation and industrialization of traditional medical knowledge.

Moreover, the limitations of the current patent system in protecting ancient classical formulations or “Jingfang” of TCM—highlight the need to prioritize the protection of such formulas as a key area for expanding the system’s coverage. According to Paragraph 2, Article 30 of the *Law of the People’s Republic of China on TCM*, “Jingfang” refers to prescriptions recorded in classical TCM texts that continue to enjoy widespread clinical use, have demonstrated therapeutic efficacy, and exhibit distinct characteristics and advantages.

Many MAHs and manufacturers view classical formulations a primary source of innovation for proprietary Chinese medicines. However, since these formulas constitute traditional public knowledge, they typically fail to meet the “novelty” requirement necessary for patent protection. Even when adaptations are made—through approaches such as “new preparations of established drugs” or “new uses of existing drugs”—most resulting formulations can still be traced back to their classical origins, rendering patent applications highly vulnerable to rejection.

Considering the substantial market potential and industrial value of classical formulations, there is a pressing need to establish robust institutional mechanisms to safeguard them. In response, the TCMVPS has expanded its coverage to include classical formulations, aligning with the reforms introduced by the NMPA in the *Announcement on Classification and Documentation Requirements for Traditional Chinese Medicine Registration* (No. 68, 2020).

This announcement established a new and independent registration pathway specifically for classical formulations, clearly defining them as *Category 3.1 new TCMs*, i.e., “compound preparations of ancient classical prescription TCMs.” Prior to this reform, these formulations were often categorized under “TCMs with existing national standards” or similar classifications, which treated them more like generics and offered unclear protection and market exclusivity. In contrast, the 2020 revision recognizes these formulations as legally new drugs, simplifying the approval process while providing a clearer and more valuable legal status, thereby laying a solid foundation for future market protection and safeguarding of rights. These institutional adjustments have effectively facilitated sustained growth in the number of protected TCM products while simultaneously strengthening the practical foundation, credibility, and enforceability of the TCMVPS.

Specific improvement measures may include:

Establishing a dedicated registration pathway for classical formulations. Drawing on models such as the European Union’s *Traditional Herbal Medicinal Products Directive* (Directive 2004/24/EC). Under this system, applicants would be required to provide historical clinical usage data (sourced from TCM texts or modern clinical studies), along with contemporary pharmacological evidence—including animal experiments and real-world research. Following expert review, eligible preparations would be designated as Class II protected varieties, granting enterprises exclusive market rights for a defined period, during which other manufacturers would be prohibited from producing identical formulations. Rights holders would be obligated to periodically update efficacy data to maintain their protected status.

Strengthen linkage between efficacy verification and process innovation Develop technical evaluation guidelines for process innovation, encouraging MAHs to apply advanced techniques to improve classical formulations. This framework would help shift the TCMVPS from an “administrative monopoly” model toward a “value-driven exclusivity” approach, emphasizing efficacy and market-oriented innovation, thereby ensuring that both the therapeutic value and methodological advancement of classical formulations are adequately protected.

Re-evaluating classical formulations post-market. To balance public interest with industry incentives, the efficacy and safety of classical formulations should be continuously assessed. Real-world evidence can be used to track therapeutic outcomes, and data disclosure should be required to maintain protected status, effectively balancing the public knowledge aspect of classical formulations with the need for practical efficacy protection.

#### Enhancing protection mechanisms: harmonizing the TCMVPS with the drug registration system

In practice, a notable critique of the TCMVPS is its potential to create institutional inertia. During the protection period, rights holders enjoy a degree of market exclusivity, which can unintentionally reduce their motivation to pursue ongoing research and development, such as expanding therapeutic indications or improving production processes (Jiang et al., 2020). If such incentives wane, the system risks deviating from its original purpose, potentially slowing sustained innovation in proprietary Chinese medicines and affecting competitive balance within the market (Li, 2019). This highlights the tension between administrative protection and the continuous advancement of technology in the TCM industry.

To facilitate comprehensive evaluation of production processes, quality standards, and post-market adverse reaction monitoring, China’s NMPA implemented the *Measures for Drug Registration* on July 1, 2020 (NMPA, 2020). Under these Measures, drug registration certificates are valid for 5 years. Manufacturers or MAHs must submit re-registration applications at least 6 months before certificate expiration, demonstrating that the drug continues to meet safety, efficacy, and quality control criteria. Starting January 1, 2025, the re-registration process was further simplified, following the *Announcement on Re-registration Procedures and Documentation Requirements for Domestically Produced Drugs* issued on October 11, 2024 (NMPA, 2024).

As a result, TCM products protected under the TCMVPS remain subject to re-registration throughout their protection period. To maintain market advantages and exclusivity, rights holders need to actively update data on expanded indications, adverse reaction monitoring, and other relevant metrics. However, classical formulations face greater challenges in meeting re-registration requirements compared with modern pharmaceuticals supported by extensive clinical trial data (Luo et al., 2021; Hu et al., 2025).

Although these formulas have been applied in clinical practice for many years, historical usage alone does not fully satisfy the systematic safety and efficacy data required by modern regulatory standards. The complexity of TCM formulations and the inherent variability in traditional production processes, as mentioned before, further complicate compliance with contemporary manufacturing and evaluation requirements (Liang et al., 2021). Consequently, classical formulations often face substantial pressure during re-registration, particularly under modern regulatory scrutiny (Li et al., 2024).

Importantly, these challenges stem not from the traditional techniques themselves, but from a misalignment between the evidence supporting these practices and the standards enforced by current regulatory frameworks. In some cases, this misalignment could result in classical formulations failing re-registration at the end of their protection period, potentially threatening continued market availability and undermining the protective intent of the TCMVPS (Liang et al., 2021).

To address these issues, the system needs to balance incentives with regulatory oversight. The TCMVPS should avoid becoming purely administrative, which could unintentionally discourage technological advancement or restrict market entry. At the same time, procedural requirements should not be so complex or stringent that they dissuade innovation.

Possible measures include:

Streamlining the re-registration process for classical formulations. For formulations already covered by the TCMVPS, a simplified re-registration pathway should be introduced. The use of real-world evidence should be encouraged (Li et al., 2023; Xia et al., 2025) so that data from routine clinical practice can be used to demonstrate safety and effectiveness.

Aligning the TCMVPS with drug-registration standards. Evaluation guidelines should be differentiated according to the specific category of classical formulations. In particular, for formulations prepared with traditional techniques, made from geo-authentic medicinal materials, or produced through methods recognized as intangible cultural heritage, the assessment criteria should be calibrated to reflect the distinctive characteristics of TCM (Li, 2019).

Additionally, a system of periodic review and dynamic exit should be established for all protected TCM products. The specific measures include:

Establishing a regular review mechanism. Rights holders should be encouraged or required to submit periodic updates, including reports on R&D progress, records of process improvements, and the latest data on efficacy and safety. An expert panel would then assess the product’s ongoing innovativeness, its clinical performance, and the robustness of its quality standards on the basis of scientific evidence and real-world clinical data.

Introducing a flexible protection term. For products showing limited innovation, the protection period or the scope of market exclusivity may be shortened or adjusted. If a manufacturer alters key production steps without authorization—such as extraction solvents, sterilization methods, or other critical parameters—and such changes raise safety concerns, the protection should be suspended or revoked.

Creating a mechanism that links information disclosure with regulatory oversight. The drug regulatory authority should incorporate the review findings and any adjustments to protection status into an official database and make this information available to companies and the public, ensuring transparency and predictability in regulatory decision-making.

## Conclusion

Many countries—especially those with rich medical, botanical, or ethnopharmacological traditions—face a common challenge when building protection regimes: how to balance community-based practices with formal IP frameworks. The evolution, limitations, and ongoing debates surrounding the TCMVPS offer a useful point of reference for jurisdictions considering field-specific or registration-based models for safeguarding traditional medical knowledge. China’s experience, although shaped by its own regulatory and cultural context, provides insights that may help other countries craft more coherent and workable approaches to protecting traditional knowledge.

By granting rights holders a period of market exclusivity, the TCMVPS offers conditional, institutional protection for techniques that have been validated through use. Yet this protection inevitably brings its own complications. Broad or lengthy protection terms may restrict market competition, raise entry barriers for other enterprises, slow technological progress, and produce inefficient allocation of industrial resources—ultimately discouraging the development of alternative or comparable products. Conversely, when qualification standards are set too high or administrative procedures become overly complex and time-consuming, the system may fail to attract innovators. In such cases, knowledge holders may be reluctant to disclose their techniques, which in turn hampers the industrialization of new traditional medicine products. Striking the right balance between encouraging innovation and maintaining a dynamic, competitive industrial environment is therefore crucial to ensuring the long-term viability of the TCMVPS. Beyond this balance, the system’s future effectiveness will depend on substantive reforms—clearer alignment with IP and drug-regulatory systems, a broader definition of protectable subject matter, and mechanisms that support continuing clinical and technological improvements.

At the same time, international legal frameworks also offer important guidance for the system’s future development. Although the TRIPS Agreement and the UPOV Convention were not designed with traditional medical knowledge in mind, their structural principles remain instructive. TRIPS’ emphasis on transparency, procedural fairness, and enforceability suggests potential directions for strengthening review standards and disclosure obligations within the TCMVPS. UPOV’s experience in defining the protectable object and setting minimum standards of protection may help refine the criteria for eligibility, the scope of rights, and the duration of protection. Thoughtfully incorporating—rather than mechanically copying—these elements could help move the TCMVPS toward a more robust, better-coordinated, and internationally compatible model.

This study has several limitations that should be acknowledged. First, because the TCMVPS is evolving within a rapidly changing regulatory environment, some of the assessments offered here may need to be revised as new rules are introduced. The absence of more detailed administrative records also means that certain aspects of internal regulatory practice—or decision-making standards that are not publicly disclosed—cannot be fully captured. Second, the analysis relies mainly on doctrinal and comparative methods. It does not incorporate interviews or stakeholder surveys, so the perspectives of industry participants, regulators, and practitioners are reflected only indirectly through secondary sources. Third, although representative cases were selected, the limited availability of complete case files makes it difficult to present the full range of contextual variations that may arise in practice.

Future research can build on these limitations by adopting empirical approaches, such as interviews with stakeholders and close observation of specific cases, to obtain a more nuanced understanding of how the TCMVPS actually operates and where institutional bottlenecks arise. Longitudinal analysis could also shed light on how the protection model influences the industrialization of TCM, including issues of knowledge transmission and commercial use. The experience of the TCMVPS offers valuable lessons for jurisdictions seeking to strike a balance between protecting traditional knowledge and aligning with modern regulatory and innovation systems. It also highlights both the opportunities and pitfalls involved in designing a sui generis protection mechanism for traditional medicine. Comparative work that examines how other countries regulate traditional medical knowledge would help identify pathways for coordination and mutual learning.
